# Sociodemographic, personal, and disease-related determinants of referral to patient-reported outcome-based follow-up of remote outpatients: a prospective cohort study

**DOI:** 10.1007/s11136-019-02407-2

**Published:** 2020-01-03

**Authors:** Liv Marit Valen Schougaard, Annette de Thurah, Jakob Christensen, Kirsten Lomborg, Helle Terkildsen Maindal, Caroline Trillingsgaard Mejdahl, Jesper Medom Vestergaard, Trine Nøhr Winding, Karin Biering, Niels Henrik Hjollund

**Affiliations:** 1 AmbuFlex/WestChronic, Occupational Medicine, University Research Clinic, Aarhus University, Gl. Landevej 61, 7400 Herning, Denmark; 2 Department of Rheumatology, Aarhus University Hospital, Aarhus, Denmark; 3 Department of Clinical Medicine, Aarhus University, Aarhus, Denmark; 4 Department of Neurology, Aarhus University Hospital, Aarhus, Denmark; 5 National Centre for Register-based Research, Department of Economics and Business Economics, Aarhus BSS, Aarhus University, Aarhus, Denmark; 6 Department of Public Health, Aarhus University, Aarhus, Denmark; 7 Department of Occupational Medicine, University Research Clinic, Regional Hospital West Jutland, Herning, Denmark; 8 Department of Clinical Epidemiology, Aarhus University Hospital, Aarhus, Denmark

**Keywords:** Patient-reported outcome measures, Ambulatory care, Outpatient clinics, hospital, Referral and consultation, Cohort study

## Abstract

**Purpose:**

We examined the association between sociodemographic, personal, and disease-related determinants and referral to a new model of health care that uses patient-reported outcomes (PRO) measures for remote outpatient follow-up (PRO-based follow-up).

**Methods:**

We conducted a prospective cohort study among outpatients with epilepsy at the Department of Neurology at Aarhus University Hospital, Denmark. Included were all persons aged ≥ 15 years visiting the department for the first time during the period from May 2016 to May 2018. Patients received a questionnaire containing questions about health literacy, self-efficacy, patient activation, well-being, and general health. We also collected data regarding sociodemographic status, labour market affiliation, and co-morbidity from nationwide registers. Associations were analysed as time-to-event using the pseudo-value approach. Missing data were handled using multiple imputations.

**Results:**

A total of 802 eligible patients were included in the register-based analyses and 411 patients (51%) responded to the questionnaire. The results based on data from registers indicated that patients were less likely to be referred to PRO-based follow-up if they lived alone, had low education or household income, received temporary or permanent social benefits, or if they had a psychiatric diagnosis. The results based on data from the questionnaire indicated that patients were less likely to be referred to PRO-based follow-up if they reported low levels of health literacy, self-efficacy, patient activation, well-being, or general health.

**Conclusion:**

Both self-reported and register-based analyses indicated that socioeconomically advantaged patients were referred more often to PRO-based follow-up than socioeconomically disadvantaged patients.

**Electronic supplementary material:**

The online version of this article (10.1007/s11136-019-02407-2) contains supplementary material, which is available to authorised users.

## Introduction

In 2019, it was estimated that two-thirds of the adult Danish population have one or multiple chronic conditions, a number that is expected to increase [1]. This increase contributes to a growing burden on the healthcare system, and to manage this challenge, several initiatives must be considered by the health authorities. One of these initiatives could be systematic use of patient-reported outcome (PRO) measures at the individual patient level in the healthcare system. PRO measures are defined as the patient’s own report on his/her health status and symptoms without interpretation by a clinician or anyone else [2]. The use of PRO measures in individual patient management has several applications; for example, it can facilitate monitoring of symptoms before and after treatment, facilitate communication between patients and clinicians, facilitate early identification of problems, and reduce unnecessary outpatient appointments for stable patients [3, 4].

The Danish PRO system, AmbuFlex, is a new model for outpatient healthcare that uses PRO measures as the basis for outpatient follow-up of patients with chronic and malignant diseases [5]. The model uses PRO measures in remote outpatient follow-up in which patients report essential information about their health status and symptoms from home instead at conventional follow-up with scheduled appointments. The PRO data are used by clinicians to decide whether a patient needs or want clinical attention, making it possible to reduce the number of unnecessary outpatient appointments [6]. In addition, the model aims to improve quality of care and promote patient-centred care. In this study, remote follow-up by using PRO measures is termed PRO-based follow-up.

Since 2012, approximately 7000 outpatients with epilepsy from five Danish neurological departments have been referred to PRO-based follow-up [5, 7]. The criteria for referral to PRO-based follow-up are not defined in a standardised guideline; instead, referral is based on the individual clinicians’ assessment of the patient together with the patients’ preferences and capabilities. The use of PRO measures in remote outpatient follow-up is a relatively new initiative that has expanded during the last 5 years in Denmark. We have not been able to identify other PRO systems that use PRO measures as the basis for follow-up of outpatients with epilepsy or in any other outpatient population, nor studies that have investigated factors associated with patients who participate in PRO-based follow-up. However, studies regarding non-response to questionnaires have found that factors associated with non-response were lower socioeconomic status [8, 9], male sex [8, 10, 11], younger age [9, 10, 12], not living with a partner [8, 10, 13], different ethnic background than Danish [10, 12], and poorer health or quality of life [8, 9]. It is therefore important that the clinicians consider these and similar aspects when deciding whether to refer a patient to PRO-based follow-up. To the best of our knowledge, no research has been conducted to explore associations between patient characteristics and referral to PRO-based follow-up.

This study aimed to identify sociodemographic, personal, and disease-related factors associated with referral to PRO-based follow-up. We hypothesised that a low level of education and household income; higher age; solo living; passive labour market participation; a low level of health literacy, self-efficacy, patient activation, well-being, and general health; and high level of co-morbidity were associated with lower probability of referral to PRO-based follow-up.

## Materials and methods

### Study design, setting and participants

We conducted a prospective cohort study among outpatients with epilepsy at the Department of Neurology at Aarhus University Hospital, Denmark. All persons aged at least 15 years visiting the department for the first time between May 2016 and May 2018 with either a diagnosis or suspicion of epilepsy were invited to participate. Eligible participants were identified in the Hospital Business Intelligence (BI) Register in the Central Denmark Region, which includes information on diagnoses classified according to the international classification of disease—version 10 (ICD-10) [14]. Data were collected every second week in patients with epilepsy (DG 40–DG409), suspicion of epilepsy (DZ033A), first time unprovoked generalised seizure (DR568E), and other non-specified seizures (DR568). A physician registers all diagnoses at hospital discharge or termination of outpatient contact. The regional registers are required by law to submit standardised data to the Danish National Patient Registry (DNPR) at least monthly. The most frequently reported measure of the validity of the records in the DNPR is the positive predictive value (PPV), defined as the proportion of patients registered with a disease who truly have the disease and usually estimated using medical record review as the reference standard to confirm the presence of disease. For the diagnosis category epilepsy in the DNPR, the PPV is estimated to be 81.4% (75.2–86.3) [15].

A research questionnaire was mailed to the study participants approximately 2 weeks after their first appointment at the department. They could choose to complete either a paper- or web-based version of the questionnaire. Non-responders received one reminder after 21 days. In addition, data on all participants including responders and non-responders were obtained from regional and national registers.

### Determinant variables

Data regarding cohabitation status, education, income, labour market affiliation, and co-morbidity were collected from available registers from Statistics Denmark. All Danish Citizens have a unique personal identification (CPR) number [16], which can be used to generate linkages between registers. Data regarding health literacy, self-efficacy, patient activation, well-being, and general health were collected by standardised questionnaires. Questionnaire data were linked with registry-based data from Statistics Denmark in January 2019 using patients’ CPR number. Table 1 presents an overview of determinant variables and data sources.

**Table 1:** Overview of determinant variables and registry and questionnaire data sources

Determinant	Data source
Age Gender	The Hospital Business Intelligence (BI) Register in Central Denmark Region [14]
Cohabitation status	The Danish Civil Registration Register (CPR) [17]
Education	The Danish Education Register [18]
Household income	Danish register on income and transfer payments [19]
Labour market affiliation	The Danish Register for Evaluation and Marginalisation (DREAM) [20]
Co-morbidity Psychiatric disease	The Danish National Patient Registry (DNPR) [21]
Well-being	WHO-Five Well-Being Index (WHO-5) [28]
General health	Short Form Health Survey 36 (SF-36) [30, 31]
Health literacy	Health Literacy Questionnaire (HLQ) [23, 24]
Self-efficacy	General Self-Efficacy Scale (GSES) [25, 26]
Patient activation	Patient Activation Measure 13 (PAM-13) [32]

#### Register data

Data on gender and age were obtained from the Hospital BI register in Central Denmark Region [14]. We used the age of the participants at the date of inclusion in the study. Age was categorised into five age groups. Cohabitation status was collected from The Danish Civil Registration System [17] the year before inclusion in the study and categorised into “Living with a partner/family” and “Living alone”. Level of education was obtained from the Danish Education Registers [18] the year before inclusion and categorised into three groups: low (< 10 years), medium (10–12 years), or high (> 12 years) educational level. Data regarding household income were collected from the Danish registers on personal income [19] the year before inclusion and categorised into low, medium, or high income according to tertiles (33.3rd and 66.6th percentile) in the study population. If cohabitation status, education, or household income data were missing in the year before inclusion, data from the previous year were used. Information about labour market affiliation was retrieved from the Danish Register for Evaluation and Marginalisation (DREAM) [20]. DREAM is a national register which contains weekly updated information about a range of temporary and permanent social benefits. Information about labour market participation was gathered for the 52-week period before the date of inclusion in the study. Based on the amount of received benefits, the participants were divided into five groups: Self-supporting (labour market or education participation): receiving social benefits for a maximum of 4 weeks; Temporary social benefits: receiving temporary social benefits for more than 4 weeks; Permanent social benefits: receiving permanent social benefits for more than 4 weeks; and Normal retirement: receiving normal retirement benefits for more than 4 weeks. Level of co-morbidity and psychiatric diseases were extracted from the DNPR [21]. The Charlson Comorbidity Index was used to categorise the participants into three groups: 0 (Low); 1–2 (Medium); > 2 (High) level of co-morbidity [22]. Psychiatric diseases (DF 00 – 99) were dichotomised into present or not within 2 years before enrolment.

#### Questionnaire data

Health literacy was measured using the Health Literacy Questionnaire (HLQ), which is a multi-dimensional questionnaire measuring a broad perception of health literacy [23, 24]. The HLQ has well-documented psychometric properties [23, 24] and consists of 44 items covering nine subscales. The following subscales were used: 4: “Social support for health”; 6: “Ability to actively engage with healthcare providers”; and 9: “Understand health information well enough to know what to do”. Subscale 4 has a four-point ordinal response options ranging from 1 “strongly disagree”, 2 “disagree”, 3 “agree” to 4 “strongly agree”. Subscales 6 and 9 have a five-point ordinal response option ranging from 1 “cannot do”, 2 “very difficult”, 3 “quite difficult”, 4 “quite easy” to 5 “very easy”. The average score across all items were estimated for each of the subscales. If items were missing, the mean score of the other items were used to estimate the scale score. The score was not estimated if more than two items were missing. Higher scores indicate a higher degree of health literacy. Subscale 4 was also dichotomised to identify participants who “strongly disagree” or “disagree” (score ≤ 2) with having social support. And subscales 6 and 9 were dichotomised to identify participants who “could not” or found it “very difficult” or “quite difficult” (scores ≤ 3) to actively engage with health care providers and understand health information. Self-efficacy was measured using the General Self-Efficacy Scale (GSE), which is a 10-item questionnaire measuring optimistic self-belief to cope with difficult tasks in life [25, 26]. The psychometric properties of the scale have been evaluated in a range of different countries and populations [27]. The 10 items have four ordinal response options ranging from 1 “not at all true”, 2 “hardly true”, 3 “moderately true” to 4 “exactly true”. The GSE score ranges from 10 to 40 (best). In addition, the GSE scale was dichotomised at the median cut-off point in the study population: < 30 (Low) and ≥ 30 (High). WHO-Five Well-being Index (WHO-5) is a questionnaire consisting of five positively worded items reflecting current mental well-being within the previous 2 weeks [28]. The instrument has demonstrated sufficient psychometric properties in a wide range of chronic conditions [28, 29]. Items are rated on a six-point ordinal scale ranging from 5 “all of the time”, 4 “most of the time”, 3 “more than half of the time”, 2 “less than half of the time”, 1 “some of the time” to 0 “at no time”. The score ranges from 0 to 100. Higher scores indicate a better degree of well-being, and a score below 50 indicates increased risk of depression [28]. The WHO-5 score was also dichotomised at < 50 (low) and ≥ 50 (high). The GSE and WHO-5 scores were not estimated if there were missing items. The Short Form Health Survey (SF-36) is a multi-dimensional questionnaire with eight subscales measuring different aspects of physical and mental health [30, 31]. In this study, only one single item was included: “In general, would you say your health is: excellent, very good, good, fair, or poor”. The variable was divided into three groups: “excellent/very good”, “good”, and “fair/poor”. Patient Activation Measure 13 (PAM-13) is a 13-item questionnaire measuring the aspect patient activation in health [32]. In this study, only two single items were included, which were modified from the PAM scale: “I am confident that I can tell when I need to get outpatient care” and “I am confident I can figure out solutions when new situations or problems arise with my health condition”, with the response categories: “disagree strongly”, “disagree”, “agree”, and “agree strongly”. The item responses were dichotomised into “disagree strongly/disagree” and “agree/agree strongly”.

### Outpatient follow-up

Department of Neurology at Aarhus University Hospital offers both PRO-based and conventional follow-up. In PRO-based follow-up, outpatients receive fixed-interval disease-specific questionnaires instead of in-clinic visits [5]. The questionnaire is coupled with a pre-defined color-algorithm used to determine whether the patients need clinical attention. Green color indicates no need of attention, red color indicates need of attention, and yellow color indicates that the patient might need attention [5, 7]. Clinicians assess the questionnaire responses together with other relevant data from the Electronic Health Record [5]. As of December 2019, 2110 epilepsy outpatients (approximately 50% of the entire outpatient population) are attending PRO-based follow-up at the department. In conventional follow-up, patients receive in-clinic visits or telephone consultations. A clinician together with the patient decides in each case whether to refer to PRO-based follow-up or conventional follow-up.

### Outcome

Patients were followed up from their first visit at the Department of Neurology at Aarhus University Hospital (the date of inclusion) until the event of interest: referral to PRO-based follow-up. Patients were censored at study end (after 18 months’ follow-up or in January 2019) or in the event of no further need for outpatient follow-up, emigration, or death, whichever came first. Patients referred to PRO-based follow-up are registered in the AmbuFlex-system by a clinician [5]. We used the date of this registration to define whether a patient was referred to PRO-based follow-up. The date was gathered from the AmbuFlex-database [5, 6]. The dates of other events (no further need for outpatient follow-up, emigration, or death) were obtained from the Hospital BI Register in the Central Denmark Region.

### Statistical analysis

We analysed the associations between register- and questionnaire-based determinants and the proportion of patients referred to PRO-based follow-up within 6, 12, and 18 months after the patients’ first visit at the department. Not all participants were followed for 12 and 18 months; therefore, the analyses were based on time to event by using the pseudo-value approach to examine the cumulative risk ratio (RR) at the three time points [33, 34]. In the pseudo-value approach, pseudo values are generated and used in a generalised linear regression. Death, emigration, and end of follow-up were considered competing risks in the model if they occurred before the event of interest. All estimates were reported with 95% confidence intervals. Age, gender, cohabitation status, education, and co-morbidity were included in the adjusted analyses. The confounder variables were selected a priori based on associations between these factors and questionnaire non-response in previous studies [8–13].

To manage the missing data problem, we decided to use the multiple imputation method [35]. Based on the assumption that data were missing at random, 100 complete datasets were created based on a model of all relevant variables measured in the population (Appendix 1). The robustness of the imputed model was evaluated by modifying the variables in the model. Furthermore, sensitivity analyses were performed in which we assumed that data were not missing at random; for example, if data were missing in patients with lower health literacy than expected from the imputations. For this group of patients, the imputed health literacy scores were reduced with one point corresponding to approximately one standard deviation (SD). Thereafter, the cumulative RRs at the three time points were analysed in a generalised linear regression using the pseudo-value approach.

Categorical data were presented as numbers and percentages. For normally distributed continuous data, means and SDs were presented, and for non-normally distributed questionnaire data, median and interquartile ranges were also presented. Data collected from registers were used to compare non-responders of questionnaire data with responders. All analyses were performed using STATA version 15 (Stata Corporation, College Station, Texas, USA).

## Results

### Characteristics of the study population

**Fig. 1:**
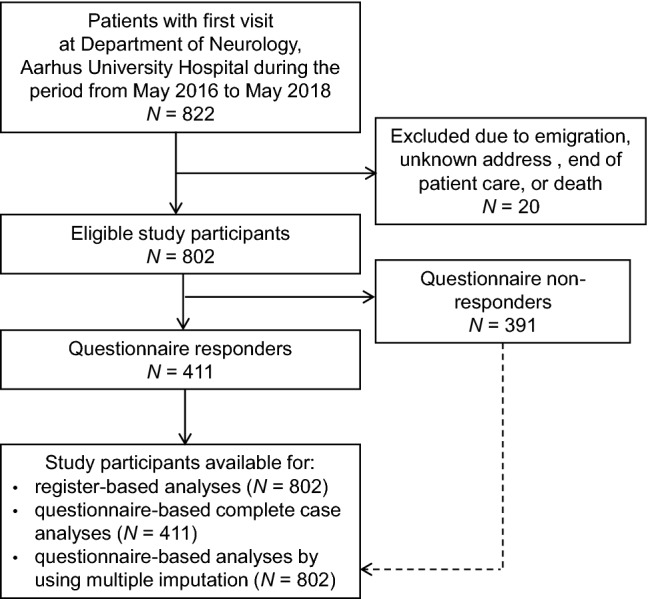
Flowchart of patients included in the study

From May 2016 to May 2018, a total of 822 patients had their first visit at the Department of Neurology at Aarhus University Hospital with either a diagnosis or suspicion of epilepsy (Fig. 1). Twenty patients were excluded due to termination of outpatient care, emigration, or death before start of follow-up, leaving 802 patients in the study. The mean age of the study population was 49.3 years (SD 21.9 years) and 52% were male (Table 2). Only 13% had a high level of co-morbidity (Charlson Comorbidity Index > 2) and 12% had a psychiatric disease diagnosis. Data were missing for three register-based variables: cohabitation status (2%), education level (7%), and household income (1%). The overall response rate was 51%, 61% for patients referred to PRO-based follow-up and 48% for patients not referred (*p *= 0.003). Questionnaire non-responders were younger (*p *< 0.001), lower educated (*p* = 0.03), more likely lived alone (*p* < 0.001), had lower household income (*p* = 0.01), and received more often temporary or social benefits (*p* < 0.001) than responders. No differences were found with regard to gender, co-morbidity, and psychiatric disease. Table 3 presents an overview of the self-reported questionnaire data from the 411 responders. There were fewer than 5% missing items for all scales expect for the GSE scale, where 9% of items were missing. The stratified data according to 18-month follow-up status indicated that patients who received conventional follow-up reported lower levels of all measured constructs than did patients referred to PRO-based follow-up.

**Table 2:** Baseline register characteristics of 802 patients and among questionnaire responders and non-responders from the Department of Neurology, Aarhus University Hospital May 2016–May 2018

	Total *n* (%)	Responders *n* (%)	Non-responders *n* (%)
	*N *= 802	*N *= 411	*N *= 391
Age, years			
15–24	168 (21)	78 (19)	90 (23)
25–39	136 (17)	46 (11)	90 (23)
40–59	187 (23)	93 (23)	94 (24)
60–69	124 (15)	76 (18)	48 (12)
70–99	187 (23)	118 (29)	69 (18)
Gender			
Female	387 (48)	204 (50)	183 (47)
Male	415 (52)	207 (50)	208 (53)
Cohabitation status			
Not living alone	492 (61)	280 (68)	212 (54)
Solo living	290 (36)	124 (30)	166 (42)
Missing	20 (2)	7 (2)	13 (3)
Education			
High (> 12 years)	135 (17)	79 (19)	56 (14)
Medium (10–12 years)	281 (35)	154 (37)	127 (32)
Low (< 10 years)	327 (41)	154 (37)	173 (44)
Missing	59 (7)	24 (6)	35 (9)
Household income^a^			
High	266 (33)		
Medium	267 (33)		
Low	261 (33)		
Missing	8 (1)		
Labour market affiliation			
Self-supporting	243 (30)	132 (32)	111 (28)
Normal retirement	234 (29)	151 (37)	83 (21)
Temporary social benefits	174 (22)	69 (17)	105 (27)
Permanent social benefits	151 (19)	59 (14)	92 (24)
Co-morbidity (Charlson index)			
Low 0	463 (58)	231 (56)	232 (59)
Medium 1–2	233 (29)	122 (30)	111 (28)
High > 2	106 (13)	58 (14)	48 (12)
Psychiatric disease			
No	703 (88)	365 (89)	338 (86)
Yes	99 (12)	46 (11)	53 (14)

^a^According to guidelines from Statistics Denmark, the distribution of household income was not reported for responders and non-responders because the missing number is below five observations

**Table 3:** Baseline self-reported characteristics and stratified according to status at 18 month follow-up^a^ among 411 patients from the Department of Neurology, Aarhus University Hospital May 2016–May 2018

	Total (%)	PRO-based follow-up	Conventional follow-up	End of outpatient care or emigrated
	*N *= 411	*n* = 113	*n* = 197	*n* = 82
Social support for health (HLQ4)				
Mean (SD)	3.3 (0.60)	3.4 (0.49)	3.2 (0.63)	3.2 (0.67)
Median (IQR)	3.4 (3.0 −3.8)	3.4 (3.0−3.8)	3.2 (3.0−3.6)	3.4 (3.0−3.8)
Missing, *n* (%)	10 (2.4)			
Ability to actively engage with healthcare providers (HLQ6)				
Mean (SD)	3.6 (0.95)	3.9 (0.83)	3.5 (0.98)	3.6 (0.99)
Median (IQR)	3.8 (3.0−4.2)	4.0 (3.4−4.4)	3.6 (3.0−4.2)	3.8 (3.1−4.3)
Missing, *n* (%)	9 (2.2)			
Understanding health information well enough to know what to do (HLQ9)				
Mean (SD)	3.6 (0.97)	3.9 (0.78)	3.5 (1.02)	3.7 (0.95)
Median (IQR)	3.8 (3.0−4.3)	4.0 (3.5−4.4)	3.8 (2.8−4.2)	4.0 (3.2−4.4)
Missing, *n* (%)	9 (2.2)			
Self-efficacy (GSE)				
Mean (SD)	27.4 (7.4)	29.0 (5.9)	26.0 (7.8)	29.5 (6.6)
Median (IQR)	29 (23−33)	30 (26−32)	27 (20−32)	30 (26−34)
Missing, *n* (%)	37 (9.0)			
Well-being (WHO-5)				
Mean (SD)	61.3 (23.9)	66.8 (21.0)	56.2 (24.9)	66.9 (20.4)
Median (IQR)	64 (48−80)	72 (56−80)	60 (36−76)	72 (52−80)
Missing, n (%)	17 (4.1)			
General health, *n* (%)				
Excellent	36 (8.8)			
Very good	94 (22.9)			
Good	149 (36.3)			
Fair	86 (20.9)			
Poor	33 (8.0)			
Missing	13 (3.1)			
Patient activation^b^, *n* (%)				
Disagree strongly	31 (7.5)			
Disagree	102 (24.8)			
Agree	170 (41.4)			
Agree strongly	94 (22.9)			
Missing	14 (3.4)			
Patient activation^c^, *n* (%)				
Disagree Strongly	49 (11.9)			
Disagree	86 (20.9)			
Agree	190 (46.2)			
Agree strongly	74 (18.0)			
Missing	12 (2.9)			

*SD* standard deviation; *IQR* interquartile range; *HLQ* Health Literacy Questionnaire; *GSE* General Self-efficacy scale; *WHO*-*5* WHO-Five Well-being Index

^a^According to Statistics Denmark’s guidelines, the distribution of variables was not reported if a cell contained less than five observations. Among 411 patients, 19 patients had died at 18-month follow (data not shown)

^b^I am confident that I can tell when I need to get outpatient care

^c^I am confident I can figure out solutions when new situations or problems arise with my health condition

Mean follow-up time was 10.6 months (SD 6.6 months). At 6-, 12-, and 18-month follow-up, 139, 173, and 185 patients had been referred to PRO-based follow-up, 95, 129, and 172 patients were no longer visiting the outpatient clinic, and 26, 43, and 52 patients had died, respectively. Fewer than 5 patients had emigrated at all three time points.

### Register determinants of referral to PRO-based follow-up

The cumulative risk ratios of referral to PRO-based follow-up 6, 12, and 18 months after the patients’ first visit at the department in relation to register determinants are presented in Table 4. At all three time points, a decreased adjusted risk of referral to PRO-based follow-up was found among patients who lived alone and patients with low education or household income. At 18-month follow-up, a decreased adjusted risk was also found in patients with temporary or permanent social benefits and patients with a psychiatric diagnosis. Further, at 18-month follow-up, men were more likely to be referred to PRO-based follow-up than women. At 12-month follow-up, a decreased adjusted risk of referral was found in patients with a medium level of co-morbidity compared to a low level of co-morbidity; however, no differences were found at 6- or 18-month follow-up. No differences were found between age groups.

**Table 4:** Risk ratio (RR) of referral to PRO-based follow-up 6, 12, and 18 months after the first visit at Department of Neurology, Aarhus University Hospital according to register determinants (*N *= 802)

	6-month follow-up		12-month follow-up		18-month follow-up	
	Crude RR	Adjusted RR^a^	Crude RR	Adjusted RR^a^	Crude RR	Adjusted RR^a^
Age, years						
15–24	Ref	Ref	Ref	Ref	Ref	Ref
25–39	1.04 (0.63–1.70)	0.99 (0.57–1.71)	0.91 (0.59–1.40)	0.85 (0.54–1.36)	0.90 (0.59–1.36)	0.84 (0.54–1.30)
40–59	1.08 (0.68–1.71)	0.98 (0.58–1.65)	1.02 (0.69–1.50)	0.94 (0.60–1.46)	1.01 (0.70–1.47)	0.92 (0.60–1.40)
60–69	1.08 (0.65–1.80)	0.77 (0.43–1.37)	0.98 (0.63–1.52)	0.83 (0.50–1.37)	0.85 (0.55–1.30)	0.73 (0.45–1.19)
70–99	1.01 (0.64–1.60)	0.92 (0.52–1.62)	0.90 (0.60–1.34)	1.06 (0.62–1.78)	0.84 (0.57–1.24)	0.95 (0.58–1.58)
Gender						
Female	Ref	Ref	Ref	Ref	Ref	Ref
Male	1.32 (0.97–1.80)	1.35 (0.95–1.92)	1.29 (0.99–1.69)	1.28 (0.96–1.69)	1.39 (1.07–1.81)	1.36 (1.03–1.79)
Cohabitation status						
Living with a partner/family	Ref	Ref	Ref	Ref	Ref	Ref
Living alone	0.61 (0.43–0.86)	0.60 (0.41–0.89)	0.55 (0.40–0.75)	0.55 (0.39–0.78)	0.58 (0.43–0.79)	0.63 (0.45–0.89)
Education						
High (> 12 years)	Ref	Ref	Ref	Ref	Ref	Ref
Medium (10–12 years)	1.03 (0.70–1.51)	0.97 (0.66–1.46)	1.25 (0.88–1.78)	1.22 (0.86–1.74)	1.20 (0.85–1.69)	1.15 (0.82–1.62)
Low (< 10 years)	0.53 (0.34–0.81)	0.46 (0.28–0.75)	0.65 (0.44–0.96)	0.62 (0.41–0.94)	0.69 (0.47–1.01)	0.65 (0.44–0.97)
Household income						
High	Ref	Ref	Ref	Ref	Ref	Ref
Medium	0.46 (0.32–0.67)	0.61 (0.39–0.94)	0.52 (0.38–0.71)	0.69 (0.48–1.00)	0.50 (0.37–0.68)	0.64 (0.44–0.93)
Low	0.49 (0.34–0.71)	0.59 (0.38–0.90)	0.43 (0.30–0.60)	0.51 (0.35–0.75)	0.41 (0.29–0.57)	0.47 (0.32–0.68)
Labour market affiliation						
Self-supporting	Ref	Ref	Ref	Ref	Ref	Ref
Normal retirement	0.71 (0.49–1.01)	0.69 (0.42–1.13)	0.74 (0.54–1.02)	0.86 (0.55–1.37)	0.65 (0.48–0.89)	0.77 (0.49–1.20)
Temporary social benefits	0.69 (0.47–1.03)	0.68 (0.45–1.05)	0.78 (0.55–1.09)	0.79 (0.56–1.13)	0.68 (0.49–0.95)	0.69 (0.49–0.96)
Permanent social benefits	0.38 (0.22–0.67)	0.55 (0.29–1.04)	0.44 (0.27–0.71)	0.60 (0.35–1.02)	0.39 (0.24–0.62)	0.51 (0.31–0.84)
Co-morbidity (Charlson Index)						
Low 0	Ref	Ref	Ref	Ref	Ref	Ref
Medium 1–2	0.79 (0.54–1.14)	0.73 (0.48–1.12)	0.72 (0.52–1.00)	0.69 (0.46–0.97)	0.74 (0.54–1.01)	0.74 (0.52–1.04)
High > 2	1.09 (0.72–1.66)	1.08 (0.68–1.71)	0.88 (0.60–1.29)	0.89 (0.60–1.33)	0.78 (0.53–1.14)	0.82 (0.55–1.23)
Psychiatric disease						
No	Ref	Ref	Ref	Ref	Ref	Ref
Yes	0.65 (0.37–1.15)	0.75 (0.42–1.36)	0.47 (0.26–0.85)	0.55 (0.30–1.01)	0.44 (0.24–0.82)	0.50 (0.27–0.93)

Numbers in round brackets are 95% confidence intervals (CIs). The estimated RRs and 95% CIs were obtained after multiple imputations in a generalised linear regression using the pseudo-value approach

^a^Mutual adjusted for age, gender, cohabitation status, education, and co-morbidity

### Questionnaire determinants of referral to PRO-based follow-up

The cumulative risk ratios of referral to PRO-based follow-up 6, 12, and 18 months after the patients’ first visit at the department in relation to questionnaire determinants are presented in Tables 5 and 6. Patients who reported a low level of perceived confidence regarding to figure out solutions or problems related to their health condition had a decreased adjusted risk of referral to PRO-based follow-up at all three time points. At 12- and 18-month follow-up, a decreased adjusted risk was found in patients with low health literacy (HLQ 9), well-being, or general health, and in patients who reported a low level of perceived confidence to decide their need for outpatient care. At 18-month follow-up, a decreased adjusted risk of referral was also found in patients with low self-efficacy. No adjusted differences were found in health literacy (HLQ 4 and HLQ 6). The questionnaire scale scores were also analysed (Table 6). In the adjusted analyses, we found that a one-unit increase in mean scale scores of HLQ 6 and 9 increased the risk of referral to PRO-based follow-up at all three time points. Similarly, at 12- and 18-month follow-up, one-unit increase in mean scale scores of HLQ 4 and WHO-5 also increased the risk of referral to PRO-based follow-up. One-unit increase in mean scale score of GSE increased the risk of referral at 18-month follow-up.

**Table 5:** Risk ratio (RR) of referral to PRO-based follow-up 6, 12, and 18 months after the first visit at Department of Neurology, Aarhus University Hospital according to questionnaire determinants (*N *= 802)

	6-month follow-up		12-month follow-up		18-month follow-up	
	Crude RR	Adjusted RR^a^	Crude RR	Adjusted RR^a^	Crude RR	Adjusted RR^a^
Social support for health (HLQ4)						
High (> 2)	Ref	Ref	Ref	Ref	Ref	Ref
Low (≤ 2)	0.47 (0.09–2.44)	0.43 (0.07–2.48)	0.43 (0.09–2.02)	0.40 (0.08–2.06)	0.38 (0.07–2.02)	0.40 (0.07–2.20)
Ability to actively engage with healthcare providers (HLQ6)						
High (> 3)	Ref	Ref	Ref	Ref	Ref	Ref
Low (≤ 3)	0.60 (0.37–0.98)	0.73 (0.42–1.28)	0.55 (0.35–0.88)	0.63 (0.38–1.05)	0.55 (0.35–0.89)	0.64 (0.39–1.05)
Understanding health information well enough to know what to do (HLQ9)						
High (> 3)	Ref	Ref	Ref	Ref	Ref	Ref
Low (≤ 3)	0.42 (0.24–0.75)	0.53 (0.28–1.01)	0.41 (0.23–0.71)	0.48 (0.26–0.87)	0.40 (0.23–0.69)	0.45 (0.25–0.82)
Self-efficacy (GSE)						
High (≥ 30)	Ref	Ref	Ref	Ref	Ref	Ref
Low (< 30)	0.61 (0.42–0.89)	0.69 (0.46–1.04)	0.63 (0.46–0.88)	0.73 (0.52–1.03)	0.60 (0.43–0.84)	0.69 (0.49–0.98)
Well-being (WHO-5)						
High (≥ 50)	Ref	Ref	Ref	Ref	Ref	Ref
Low (< 50)	0.67 (0.43–1.03)	0.73 (0.45–1.18)	0.54 (0.36–0.82)	0.59 (0.38–0.91)	0.50 (0.33–0.78)	0.55 (0.35–0.86)
General health						
Excellent/very good	Ref	Ref	Ref	Ref	Ref	Ref
Good	1.02 (0.67–1.54)	1.07 (0.69–1.65)	0.82 (0.56–1.19)	0.88 (0.60–1.30)	0.71 (0.50–1.02)	0.76 (0.52–1.12)
Fair/poor	0.61 (0.36–1.02)	0.72 (0.41–1.25)	0.47 (0.29–0.75)	0.57 (0.34–0.94)	0.38 (0.24–0.61)	0.46 (0.28–0.76)
Patient activation^b^						
Agree strongly/agree	Ref	Ref	Ref	Ref	Ref	Ref
Disagree strongly/disagree	0.55 (0.36–0.86)	0.64 (0.38–1.07)	0.57 (0.38–0.85)	0.65 (0.42–0.99)	0.51 (0.34–0.76)	0.57 (0.38–0.88)
Patient activation^c^						
Agree strongly/agree	Ref	Ref	Ref	Ref	Ref	Ref
Disagree strongly/disagree	0.54 (0.34–0.86)	0.58 (0.34–0.97)	0.56 (0.37–0.86)	0.59 (0.37–0.93)	0.50 (0.33–0.76)	0.54 (0.35–0.83)

*HLQ* Health Literacy Questionnaire; *GSE* General Self-efficacy scale; *WHO*-*5* WHO-Five Well-being Index

Numbers in round brackets are 95% confidence intervals (CIs).The estimated RRs and 95% CIs were obtained after multiple imputations in a generalised linear regression using the pseudo-value approach

^a^Adjusted for age, gender, cohabitation status, education, and co-morbidity

^b^I am confident that I can tell when I need to get outpatient care

^c^I am confident I can figure out solutions when new situations or problems arise with my health condition

**Table 6:** Risk ratio (RR) of referral to PRO-based follow-up 6, 12, and 18 months after the first visit at Department of Neurology, Aarhus University Hospital according to questionnaire scale scores (*N *= 802)

	6-month follow-up		12-month follow-up		18-month follow-up	
	Crude RR	Adjusted RR^a^	Crude RR	Adjusted RR^a^	Crude RR	Adjusted RR^a^
Social support for health (HLQ4 score)	1.40 (1.04–1.87)	1.35 (0.99–1.87)	1.38 (1.06–1.80)	1.31 (1.00–1.72)	1.49 (1.13–1.97)	1.40 (1.05–1.87)
Ability to actively engage with healthcare providers (HLQ6 score)	1.35 (1.12–1.64)	1.26 (1.00–1.59)	1.38 (1.16–1.65)	1.32 (1.09–1.61)	1.40 (1.18–1.66)	1.34 (1.12–1.61)
Understanding health information well enough to know what to do (HLQ9 score)	1.39 (1.15–1.67)	1.30 (1.04–1.63)	1.35 (1.16–1.57)	1.30 (1.09–1.54)	1.40 (1.19–1.65)	1.36 (1.13–1.62)
Self-efficacy (GSE score)	1.03 (1.01–1.05)	1.02 (0.99–1.05)	1.03 (1.01–1.05)	1.02 (0.99–1.04)	1.03 (1.01–1.06)	1.03 (1.00–1.05)
Well-being Index (WHO-5 score)	1.01 (1.00–1.02)	1.01 (0.99–1.01)	1.01 (1.00–1.02)	1.02 (1.00–1.02)	1.01 (1.01–1.02)	1.01 (1.00–1.02)

*HLQ* Health Literacy Questionnaire; *GSE* General Self-efficacy scale; *WHO*-*5* WHO-Five Well-being Index

Numbers in round brackets are 95% confidence intervals (CIs).The estimated RRs and 95% CIs were obtained after multiple imputations in a generalised linear regression using the pseudo-value approach

^a^Adjusted for age, gender, cohabitation status, education, and co-morbidity

### Other analyses

The results based on multiple imputations were comparable to the original raw data analyses (Appendix 2). The imputations did not change the estimates markedly. In addition, the results were not affected by modifying the variables in the multiple imputation model. The sensitivity analyses of the health literacy scores did not alter the results noticeably (Appendix 3).

## Discussion

This study showed that several sociodemographic, personal, and disease-related factors play a role in referral to PRO-based follow-up. Patients were less likely to be referred to PRO-based follow-up if they lived alone or had low education or household income, if they received temporary or permanent social benefits, or if they had a psychiatric diagnosis. Furthermore, we found that patients were less likely to be referred to PRO-based follow-up if they reported a low level of health literacy, self-efficacy, patient activation, well-being, or general health.

A shift toward more active involvement of patients in chronic disease care management is taking place in the healthcare system, characterised by productive interactions between patients and health care providers [36, 37]. These interactions do not necessarily require face-to-face visits [37]. In addition, management of chronic diseases is shifting from the clinic to the patients’ homes [38]. PRO-based follow-up and telephone consultations provided by nurses [39] are examples of care at a distance in epilepsy outpatient follow-up. John et al. argue that telephone follow-up could replace traditional scheduled appointments unless there is a clear clinical need; for example, if the patient is considered vulnerable [39].

Patients with chronic diseases need the skills, confidence, and information necessary to make best use of their involvement in self-management [37]. They must cope with increasing treatment workload, e.g. taking medication, reading information, and making lifestyle changes. The treatment workload should balance the patients’ capacity [38, 40]. Low capacity concurrent with high workload may diminish self-management, adherence to treatment, and health outcomes [38]. Increased risk of depression, poorer quality of life, and more social stigma have been found in patients with seizures compared to patients with no seizures [41, 42], which supports the need for tighter follow-up strategy in patients with severe epilepsy than in patients with stable disease. PRO-based follow-up aims to optimise the healthcare resources, as patients with no need of clinical attention are not routinely seen in-clinic. Hence, resources can be used to respond rapidly to patients with a high symptom burden. A qualitative study found that clinicians experienced that problems were more complex in the patients seen in-clinic after implementation of PRO-based follow-up [43].

We found that PRO-based follow-up is offered to a selected group of socioeconomically advantaged patients. The goal has never been to refer the whole outpatient population. The decision must be based on the patient’s preferences and clinical profile. In PRO-based follow-up, patients fill in scheduled questionnaires during follow-up; hence, we considered it relevant to consider factors related to questionnaire non-response. We found that lower sociodemographic status was associated with a decreased probability of referral to PRO-based follow-up. This finding is supported by studies regarding questionnaire non-response [8, 9]. A Danish study among patients with endometrial cancer found that well-educated patients more often sought medical attendance if symptoms of recurrence occurred than did less educated patients [44]. We also found an association between a lower degree of self-reported patient activation and non-referral to PRO-based follow-up. A recent study of patients referred to advanced heart failure therapy used the PAM scale to measure the degree of patient activation [45]. In accordance with our findings, they also found that those not selected for therapy were more likely to have lower patient activation than those who were selected [45].

A qualitative study has documented a variation in patients’ preferences for being active and taking responsibility in PRO-based follow-up, as some patients experienced a lack of confidence in their own capability to participate [46]. In addition, a study regarding the clinician perspective indicated that some clinicians had concerns regarding some patients’ capability to participate in PRO-based follow-up, even though the patient had already been referred [43]. We cannot rule out that some clinicians were more reluctant to introduce PRO-based follow-up and did not refer all relevant patients. On the other hand, patients could also have been referred without having the skills or confidence to participate. Preferably, referral to PRO-based follow-up should be based on a shared decision between the patient and the clinician in which both advantages and disadvantages are discussed. This may strengthen the patients’ expectations and willingness to participate in PRO-based follow-up and prevent non-response and dropout during PRO-based follow-up.

A high level of health literacy skills has been associated with health-promoting behaviours and better health outcomes in relation to self-reported health status, dietary habits, physical activity, smoking, alcohol consumption, and glycaemic control of diabetes [47–49]. We found that lower health literacy was associated with a decreased probability of referral to PRO-based follow-up. Although our finding indicated that clinicians are aware of the patients’ health literacy before referring a patient to PRO-based follow-up, a future focus should be on how healthcare services can be supportive toward vulnerable patients. Follow-up care for patients with a low level of health literacy may need to be more clinician-driven, clinicians needing to concentrate on increasing the health literacy level to prevent diminished participation in activities in relation to disease prevention or progression. PRO measures during follow-up of vulnerable patients may be difficult because some patients with epilepsy have cognitive disabilities and are not capable of filling in a questionnaire on their own. For this reason, the department has developed a PRO-based proxy solution in which a relative or social worker fills in the questionnaire on behalf of the patient. As of December 2019, 85 patients are attending the proxy solution at the department.

This is a large Danish prospective cohort study among outpatients with epilepsy in which register data from all the included participants were used. The study population was identified in the Hospital BI Register in the Central Denmark Region by using four selected ICD-10 codes. The selection of codes was based on a random sample of epilepsy outpatients attending PRO-based follow-up in 2015 and advice from a neurologist at the department. The four codes covered 96.4% of the diagnoses given to the patients in the random sample. Although the PPV of epilepsy diagnoses was high in the DNPR, the completeness of the four ICD-10 codes in the register is unknown; hence, there may have been patients with epilepsy or suspicion of epilepsy at the department who were not recorded in the database. However, lack of registration in the DNPR or the BI register is considered to be random; thus, the risk of bias related to selection of the study population is considered to be limited. Information bias related to registry-based analyses is also considered to be limited. Misclassification of data from registers is most likely random since the data collection is based on administrative requirements. Any potential bias would be non-differential as any missing data or misclassification took place before the event of interest (PRO-based follow-up).

Questionnaire non-response could potentially bias the estimates in both directions. The response rate was only 51% and non-responders differed from responders, as they were younger, lower educated, and received more temporary social benefits. Questionnaire non-responders were also related to the event of interest as they were less likely to be referred to PRO-based follow-up. We assumed that data were missing at random, but it is not possible to prove this. Because data may not have been missing at random, we assumed in the sensitivity analyses that the scores of HLQ were lower than the imputed values for patients with missing HLQ scores. However, the results did not change noticeably. The risk of information bias should also be considered for self-reported data. The questionnaire response took place before referral to PRO-based follow-up; thus, any misclassification of self-reported information most likely resulted in non-differential bias. We decided to dichotomise the questionnaire scale scores into a low or high level of the construct of interest to better interpret and present the results. However, dichotomisation of continuous variables entails loss of information and statistical power [50]. As can be seen in the wide confidence intervals of HLQ4 in Table 5, few participants reported ‘disagree or disagree strongly’ to the questions regarding social support for health. Thus, in this study, the continuous HLQ4 scale contained more information and statistical power than the dichotomised form.

The study population was recruited from only one neurologic department in Denmark. The department is a large, highly specialised department with a large number of epilepsy patients compared to minor regional hospitals. The department was also the first department in Denmark to offer PRO-based follow-up for outpatients with epilepsy, and has a long experience with the use of PRO measures in remote outpatient follow-up. However, despite this being a single-unit study, we expect that the results may be generalised to outpatients with epilepsy and perhaps also to other patient populations with a chronic or long-term condition.

## Conclusion

PRO-based follow-up has been used in Denmark since 2012, and since then, approximately 7000 epilepsy outpatients have been referred to PRO-based follow-up at five hospitals. Several sociodemographic, personal, and disease-related factors play a role in referral to PRO-based follow-up. Both register and questionnaire data were consistent and indicated that socioeconomically advantaged patients were more likely to be referred to PRO-based follow-up than less socioeconomically advantaged patients. Further research should explore how health care services to a larger extent can be supportive towards less advantaged patients.

## Electronic supplementary material

Supplementary material 1 (PDF 518 kb)Below is the link to the electronic supplementary material
