# Supplementary material for: Sociodemographic, personal, and disease-related determinants of referral to patient-reported outcome-based follow-up of remote outpatients: a prospective cohort study
Source: Qual Life Res. 2020 Jan 3;29(5):1335–47. doi: 10.1007/s11136-019-02407-2 (PMC7190685; doi:10.1007/s11136-019-02407-2)
Supplement: Supplementary file 1 — Supplementary material 1 (PDF 518 kb)Below is the link to the electronic supplementary material [file 11136_2019_2407_MOESM1_ESM.pdf]

TITLE: Sociodemographic, personal, and disease-related determinants of referral to patient-reported outcome-based follow-up of remote outpatients: a prospective cohort study

AUTHORS: Liv Marit Valen Schougaard[1], Annette de Thurah[2,3], Jakob Christensen[3,4,5], Kirsten Lomborg[3], Helle Terkildsen Maindal[6], Caroline Trillingsgaard Mejdahl[1], Jesper Medom Vestergaard[7], Trine Nøhr Winding[7], Karin Biering[3,7], Niels Henrik Hjollund[1,8]

AFFILIATIONS:

[1]AmbuFlex/WestChronic, Occupational Medicine, University Research Clinic, Aarhus University, Herning, Denmark

[2] Department of Rheumatology, Aarhus University Hospital, Aarhus, Denmark

[3] Department of Clinical Medicine, Aarhus University, Aarhus, Denmark

[4] Department of Neurology, Aarhus University Hospital, Aarhus, Denmark

[5] National Centre for Register-based Research, Department of Economics and Business Economics, Aarhus BSS, Aarhus University, Denmark

[6] Department of Public Health, Aarhus University, Aarhus, Denmark

[7] Department of Occupational Medicine, University Research Clinic, Regional Hospital West Jutland, Herning, Denmark

[8] Department of Clinical Epidemiology, Aarhus University Hospital, Aarhus, Denmark

CORRESPONDING AUTHOR: Liv Marit Valen Schougaard, email: livschou@rm.dk

Appendix 1: Multiple imputation models

Based on the assumption that data were missing at random, 100 complete datasets were created based on a model (model 1) of all relevant variables measured in the population (age, gender, cohabitation status, education, household income, labour market affiliation, co-morbidity, psychiatric diseases, duration of epilepsy diagnosis, and questionnaire scores). The robustness of the imputed model was evaluated by modifying the variables in the model (model 2 and 3).

	**MODEL 1**																								
(	logit	, include(	alder	sex		udd_grp	AEKVIVADISP_13_grp_recode	dream_grp_recode		psyk		hlq_4_score	hlq_6_score	hlq_9_score	gse_score					pseudo1	pseudo2	pseudo3	))	bor_alene	///
(	ologit	, include(	alder	sex	bor_alene	udd_grp		dream_grp_recode		psyk				hlq_9_score	gse_score					pseudo1	pseudo2	pseudo3	))	AEKVIVADISP_13_grp_recode	///
(	ologit	, include(	alder	sex	bor_alene		AEKVIVADISP_13_grp_recode	dream_grp_recode	charlson_index_lpr_recode	psyk		hlq_4_score	hlq_6_score	hlq_9_score	gse_score	who5	sf_gh1h	pam_5	pam_12	pseudo1	pseudo2	pseudo3	))	udd_grp	///
(	regress	, include(	alder	sex	bor_alene	udd_grp		dream_grp_recode		psyk			hlq_6_score	hlq_9_score	gse_score	who5	sf_gh1h	pam_5	pam_12	pseudo1	pseudo2	pseudo3	))	hlq_4_score	///
(	regress	, include(	alder	sex	bor_alene	udd_grp		dream_grp_recode	charlson_index_lpr_recode	psyk	dg40_varighed_aar_ny	hlq_4_score		hlq_9_score	gse_score	who5	sf_gh1h	pam_5	pam_12	pseudo1	pseudo2	pseudo3	))	hlq_6_score	///
(	regress	, include(	alder	sex	bor_alene	udd_grp	AEKVIVADISP_13_grp_recode	dream_grp_recode	charlson_index_lpr_recode	psyk	dg40_varighed_aar_ny	hlq_4_score	hlq_6_score		gse_score	who5	sf_gh1h	pam_5	pam_12	pseudo1	pseudo2	pseudo3	))	hlq_9_score	///
(	regress	, include(	alder	sex	bor_alene	udd_grp	AEKVIVADISP_13_grp_recode	dream_grp_recode	charlson_index_lpr_recode	psyk	dg40_varighed_aar_ny	hlq_4_score	hlq_6_score	hlq_9_score		who5	sf_gh1h	pam_5	pam_12	pseudo1	pseudo2	pseudo3	))	gse_score	///
(	regress	, include(	alder	sex				dream_grp_recode	charlson_index_lpr_recode	psyk		hlq_4_score	hlq_6_score	hlq_9_score	gse_score		sf_gh1h	pam_5	pam_12	pseudo1	pseudo2	pseudo3	))	who5	///
(	ologit	, include(	alder	sex			AEKVIVADISP_13_grp_recode	dream_grp_recode	charlson_index_lpr_recode	psyk		hlq_4_score	hlq_6_score	hlq_9_score	gse_score	who5		pam_5	pam_12	pseudo1	pseudo2	pseudo3	))	sf_gh1h	///
(	ologit	, include(	alder	sex	bor_alene	udd_grp	AEKVIVADISP_13_grp_recode	dream_grp_recode	charlson_index_lpr_recode	psyk		hlq_4_score	hlq_6_score	hlq_9_score	gse_score	who5	sf_gh1h		pam_12	pseudo1	pseudo2	pseudo3	))	pam_5	///
(	ologit	, include(	alder	sex	bor_alene	udd_grp	AEKVIVADISP_13_grp_recode	dream_grp_recode	charlson_index_lpr_recode	psyk		hlq_4_score	hlq_6_score	hlq_9_score	gse_score	who5	sf_gh1h	pam_5		pseudo1	pseudo2	pseudo3	))	pam_12	///
	**MODEL 2**																								
(	logit	, include(	alder	sex		udd_grp	AEKVIVADISP_13_grp_recode	dream_grp_recode				hlq_4_score	hlq_6_score	hlq_9_score	gse_score					pseudo1	pseudo2	pseudo3	))	bor_alene	///
(	ologit	, include(	alder	sex	bor_alene	udd_grp		dream_grp_recode						hlq_9_score	gse_score					pseudo1	pseudo2	pseudo3	))	AEKVIVADISP_13_grp_recode	///
(	ologit	, include(	alder	sex	bor_alene		AEKVIVADISP_13_grp_recode	dream_grp_recode				hlq_4_score	hlq_6_score	hlq_9_score	gse_score	who5	sf_gh1h	pam_5	pam_12	pseudo1	pseudo2	pseudo3	))	udd_grp	///
(	regress	, include(	alder	sex	bor_alene	udd_grp		dream_grp_recode					hlq_6_score	hlq_9_score	gse_score	who5	sf_gh1h	pam_5	pam_12	pseudo1	pseudo2	pseudo3	))	hlq_4_score	///
(	regress	, include(	alder	sex	bor_alene	udd_grp		dream_grp_recode				hlq_4_score		hlq_9_score	gse_score	who5	sf_gh1h	pam_5	pam_12	pseudo1	pseudo2	pseudo3	))	hlq_6_score	///
(	regress	, include(	alder	sex	bor_alene	udd_grp	AEKVIVADISP_13_grp_recode	dream_grp_recode				hlq_4_score	hlq_6_score		gse_score	who5	sf_gh1h	pam_5	pam_12	pseudo1	pseudo2	pseudo3	))	hlq_9_score	///
(	regress	, include(	alder	sex	bor_alene	udd_grp	AEKVIVADISP_13_grp_recode	dream_grp_recode				hlq_4_score	hlq_6_score	hlq_9_score		who5	sf_gh1h	pam_5	pam_12	pseudo1	pseudo2	pseudo3	))	gse_score	///
(	regress	, include(	alder	sex				dream_grp_recode				hlq_4_score	hlq_6_score	hlq_9_score	gse_score		sf_gh1h	pam_5	pam_12	pseudo1	pseudo2	pseudo3	))	who5	///
(	ologit	, include(	alder	sex			AEKVIVADISP_13_grp_recode	dream_grp_recode				hlq_4_score	hlq_6_score	hlq_9_score	gse_score	who5		pam_5	pam_12	pseudo1	pseudo2	pseudo3	))	sf_gh1h	///
(	ologit	, include(	alder	sex	bor_alene	udd_grp	AEKVIVADISP_13_grp_recode	dream_grp_recode				hlq_4_score	hlq_6_score	hlq_9_score	gse_score	who5	sf_gh1h		pam_12	pseudo1	pseudo2	pseudo3	))	pam_5	///
(	ologit	, include(	alder	sex	bor_alene	udd_grp	AEKVIVADISP_13_grp_recode	dream_grp_recode				hlq_4_score	hlq_6_score	hlq_9_score	gse_score	who5	sf_gh1h	pam_5		pseudo1	pseudo2	pseudo3	))	pam_12	///
	**MODEL 3**																								
(	logit	, include(	alder	sex		udd_grp	AEKVIVADISP_13_grp_recode	dream_grp_recode		psyk										pseudo1	pseudo2	pseudo3	))	bor_alene	///
(	ologit	, include(	alder	sex	bor_alene	udd_grp		dream_grp_recode		psyk										pseudo1	pseudo2	pseudo3	))	AEKVIVADISP_13_grp_recode	///
(	ologit	, include(	alder	sex	bor_alene		AEKVIVADISP_13_grp_recode	dream_grp_recode	charlson_index_lpr_recode	psyk										pseudo1	pseudo2	pseudo3	))	udd_grp	///
(	regress	, include(	alder	sex	bor_alene	udd_grp		dream_grp_recode		psyk										pseudo1	pseudo2	pseudo3	))	hlq_4_score	///
(	regress	, include(	alder	sex	bor_alene	udd_grp		dream_grp_recode	charlson_index_lpr_recode	psyk	dg40_varighed_aar_ny									pseudo1	pseudo2	pseudo3	))	hlq_6_score	///
(	regress	, include(	alder	sex	bor_alene	udd_grp	AEKVIVADISP_13_grp_recode	dream_grp_recode	charlson_index_lpr_recode	psyk	dg40_varighed_aar_ny									pseudo1	pseudo2	pseudo3	))	hlq_9_score	///
(	regress	, include(	alder	sex	bor_alene	udd_grp	AEKVIVADISP_13_grp_recode	dream_grp_recode	charlson_index_lpr_recode	psyk	dg40_varighed_aar_ny									pseudo1	pseudo2	pseudo3	))	gse_score	///
(	regress	, include(	alder	sex				dream_grp_recode	charlson_index_lpr_recode	psyk										pseudo1	pseudo2	pseudo3	))	who5	///
(	ologit	, include(	alder	sex			AEKVIVADISP_13_grp_recode	dream_grp_recode	charlson_index_lpr_recode	psyk										pseudo1	pseudo2	pseudo3	))	sf_gh1h	///
(	ologit	, include(	alder	sex	bor_alene	udd_grp	AEKVIVADISP_13_grp_recode	dream_grp_recode	charlson_index_lpr_recode	psyk										pseudo1	pseudo2	pseudo3	))	pam_5	///
(	ologit	, include(	alder	sex	bor_alene	udd_grp	AEKVIVADISP_13_grp_recode	dream_grp_recode	charlson_index_lpr_recode	psyk										pseudo1	pseudo2	pseudo3	))	pam_12	///

Appendix 2: Original raw analyses

Risk ratio (RR) of referral to PRO-based follow-up 6, 12, and 18 months after the first visit at Department of Neurology, Aarhus University Hospital according to register determinants (*N*=802)

	6-month follow-up		12-month follow-up		18-month follow-up	
	**Crude RR**	**Adjusted RR[a]**	**Crude RR**	**Adjusted RR[a]**	**Crude RR**	**Adjusted RR[a]**
Age, years						
15−24	Ref	Ref	Ref	Ref	Ref	Ref
25−39	1.03 (0.63−1.67)	0.98 (0.58−1.66)	0.90 (0.59−1.37)	0.84 (0.53−1.31)	0.89 (0.59−1.32)	0.79 (0.51−1.24)
40−59	1.04 (0.66−1.65)	0.87 (0.52−1.44)	0.98 (0.67−1.44)	0.83 (0.54−1.28)	0.97 (0.67−1.40)	0.83 (0.55−1.25)
60−69	1.08 (0.65−1.77)	0.74 (0.42−1.30)	0.97 (0.63−1.48)	0.74 (0.45−1.21)	0.83 (0.55−1.26)	0.66 (0.40−1.06)
70−99	0.97 (0.61−1.54)	0.85 (0.48−1.49)	0.85 (0.57−1.27)	0.95 (0.57−1.61)	0.78 (0.53−1.16)	0.87 (0.52−1.43)
Gender						
Female	Ref	Ref	Ref	Ref	Ref	Ref
Male	1.32 (0.97−1.79)	1.33 (0.94−1.87)	1.29 (0.99−1.69)	1.28 (0.97−1.70)	1.39 (1.07−1.80)	1.34 (1.02−1.75)
Cohabitation status						
Living with a partner/family	Ref	Ref	Ref	Ref	Ref	Ref
Living alone	0.62 (0.44−0.87)	0.65 (0.44−0.95)	0.56 (0.41−0.76)	0.61 (0.43−0.86)	0.58 (0.43−0.79)	0.67 (0.48−0.93)
Education						
High (> 12 years)	Ref	Ref	Ref	Ref	Ref	Ref
Medium (10−12 years)	1.06 (0.72−1.55)	1.00 (0.67−1.49)	1.31 (0.91−1.87)	1.25 (0.87−1.78)	1.26 (0.88−1.78)	1.19 (0.85−1.68)
Low (< 10 years)	0.56 (0.36−0.86)	0.47 (0.29−0.76)	0.69 (0.47−1.03)	0.63 (0.41−0.96)	0.73 (0.50−1.07)	0.66 (0.44−0.98)
Household income						
High	Ref	Ref	Ref	Ref	Ref	Ref
Medium	0.46 (0.31−0.66)	0.61 (0.39−0.96)	0.52 (0.38−0.71)	0.71 (0.48−1.04)	0.49 (0.36−0.67)	0.67 (0.46−0.97)
Low	0.50 (0.35−0.72)	0.65 (0.43−0.99)	0.44 (0.32−0.62)	0.59 (0.41−0.85)	0.43 (0.31−0.59)	0.52 (0.36−0.75)
Labour market affiliation						
Self-supporting	Ref	Ref	Ref	Ref	Ref	Ref
Normal retirement	0.69 (0.48−1.00)	0.71 (0.43−1.19)	0.71 (0.52−0.98)	0.94 (0.57−1.54)	0.63 (0.46−0.86)	0.82 (0.51−1.34)
Temporary social benefits	0.69 (0.46−1.02)	0.68 (0.45−1.03)	0.76 (0.54−1.07)	0.79 (0.55−1.11)	0.67 (0.49−0.94)	0.66 (0.47−0.93)
Permanent social benefits	0.38 (0.21−0.66)	0.52 (0.27−0.99)	0.43 (0.26−0.70)	0.56 (0.32−0.98)	0.39 (0.24−0.62)	0.48 (0.28−0.82)
Co-morbidity (Charlson Index)						
Low 0	Ref	Ref	Ref	Ref	Ref	Ref
Medium 1−2	0.77 (0.53−1.12)	0.73 (0.48−1.11)	0.70 (0.50−0.97)	0.67 (0.46−0.97)	0.71 (0.52−0.97)	0.73 (0.51−1.03)
High > 2	1.02 (0.66−1.59)	0.98 (0.61−1.57)	0.79 (0.52−1.20)	0.80 (0.51−1.24)	0.68 (0.44−1.05)	0.72 (0.45−1.13)

Psychiatric disease						
No	Ref	Ref	Ref	Ref	Ref	Ref
Yes	0.65 (0.37−1.15)	0.77 (0.43−1.38)	0.48 (0.27−0.86)	0.57 (0.31−1.04)	0.46 (0.25−0.83)	0.52 (0.28−0.96)

Numbers in round brackets are 95% confidence intervals (CIs).The estimated RRs and 95% CIs were obtained after multiple imputations in a generalised linear regression using the pseudo-value approach.

[a] Mutual adjusted for age, gender, cohabitation status, education, and co-morbidity

Risk ratio (RR) of referral to PRO-based follow-up 6, 12, and 18 months after the first visit at Department of Neurology, Aarhus University Hospital according to questionnaire determinants (*N*=411)

	**6-month follow-up**		**12-month follow-up**		**18-month follow-up**	
	**Crude RR**	**Adjusted RR**[a]	**Crude RR**	**Adjusted RR**[a]	**Crude RR**	**Adjusted RR**[a]
Social support for health (HLQ4)						
High (> 2)	Ref	Ref	Ref	Ref	Ref	Ref
Low (≤ 2)	0.57 (0.13−2.58)	0.32 (0.04−2.45)	0.50 (0.12−2.03)	0.32 (0.04−2.32)	0.45 (0.10−1.93)	0.34 (0.05−2.51)
Ability to actively engage with healthcare providers (HLQ6)						
High (> 3)	Ref	Ref	Ref	Ref	Ref	Ref
Low (≤ 3)	0.70 (0.42−1.19)	0.78 (0.42−1.45)	0.64 (0.39−1.04)	0.68 (0.38−1.23)	0.64 (0.39−1.05)	0.69 (0.39−1.23)
Understanding health information well enough to know what to do (HLQ9)						
High (> 3)	Ref	Ref	Ref	Ref	Ref	Ref
Low (≤ 3)	0.41 (0.21−0.82)	0.48 (0.23−0.98)	0.32 (0.16−0.65)	0.35 (0.17−0.74)	0.33 (0.17−0.66)	0.35 (0.17−0.73)
Self-efficacy (GSE)						
High (≥ 30)	Ref	Ref	Ref	Ref	Ref	Ref
Low (< 30)	0.63 (0.43−0.93)	0.73 (0.48−1.13)	0.64 (0.45−0.90)	0.72 (0.49−1.07)	0.64 (0.46−0.90)	0.70 (0.49−1.01)
Well-being (WHO-5)						
High (≥ 50)	Ref	Ref	Ref	Ref	Ref	Ref
Low (< 50)	0.71 (0.45−1.12)	0.78 (0.46−1.30)	0.57 (0.37−0.89)	0.62 (0.38−1.02)	0.50 (0.32−0.79)	0.57 (0.36−0.92)
General health						
Excellent/ Very good	Ref	Ref	Ref	Ref	Ref	Ref
Good	1.21 (0.80−1.82)	1.20 (0.76−1.87)	0.99 (0.70−1.41)	0.97 (0.63−1.48)	0.87 (0.62−1.21)	0.84 (0.57−1.23)
Fair/ Poor	0.66 (0.39−1.14)	0.73 (0.42−1.27)	0.53 (0.32−0.86)	0.61 (0.36−1.02)	0.43 (0.26−0.71)	0.48 (0.29−0.82)
Patient activation [b]						
Agree Strongly/ Agree	Ref	Ref	Ref	Ref	Ref	Ref
Disagree Strongly/ Disagree	0.54 (0.33−0.87)	0.54 (0.29−1.02)	0.57 (0.37−0.86)	0.59 (0.35−0.99)	0.49 (0.32−0.76)	0.50 (0.30−0.85)
Patient activation [c]						
Agree Strongly/ Agree	Ref	Ref	Ref	Ref	Ref	Ref
Disagree Strongly/ Disagree	0.53 (0.33−0.87)	0.51 (0.29−0.91)	0.52 (0.34−0.81)	0.52 (0.31−0.89)	0.46 (0.29−0.72)	0.45 (0.26−0.77)

Abbreviations HLQ: Health Literacy Questionnaire; GSE: General Self-efficacy scale; WHO-5: WHO-Five Well-being Index

Numbers in round brackets are 95% confidence intervals (CIs).The estimated RRs and 95% CIs were obtained after multiple imputations in a generalised linear regression using the pseudo-value approach. [a] Adjusted for age, gender, cohabitation status, education, and co-morbidity

[b] I am confident that I can tell when I need to get outpatient care

[c] I am confident I can figure out solutions when new situations or problems arise with my health condition

Appendix 3: Sensitivity analysis

We assumed that self-reported health literacy missing data were lower than expected from the imputed dataset. For patients with missing self-reported health literacy data, health literacy scores were reduced with one point corresponding to approximately one standard deviation. Subsequently, the cumulative risk ratio (RR) was analysed by a generalised linear regression using the pseudo-value approach at the three time points.

Risk ratio (RR) of referral to PRO-based follow-up 6, 12, and 18 months after the first visit at Department of Neurology, Aarhus University Hospital according to self-reported health literacy (*N*=802)

	**6-month follow-up**		**12-month follow-up**		**18-month follow-up**	
	**Crude RR**	**Adjusted RR[a]**	**Crude RR**	**Adjusted RR[a]**	**Crude RR**	**Adjusted RR[a]**
Social support for health (HLQ4 score)	1.45 (1.19−1.77)	1.32 (1.04−1.67)	1.38 (1.16−1.65)	1.24 (1.02−1.51)	1.42 (1.18−1.71)	1.30 (1.06−1.59)
Ability to actively engage with healthcare providers (HLQ6 score)	1.40 (1.19−1.64)	1.27 (1.04−1.55)	1.38 (1.20−1.58)	1.28 (1.10−1.49)	1.39 (1.21−1.58)	1.30 (1.13−1.51)
Understanding health information well enough to know what to do (HLQ9 score)	1.42 (1.22−1.65)	1.30 (1.08−1.56)	1.35 (1.19−1.54)	1.26 (1.09−1.46)	1.38 (1.21−1.57)	1.31 (1.13−1.52)

Abbreviations HLQ: Health Literacy Questionnaire; GSE: General Self-efficacy scale; WHO-5: WHO-Five Well-being Index
Numbers in round brackets are 95% confidence intervals (CIs).The estimated RRs and 95% CIs were obtained after multiple imputations in a generalised linear regression using the pseudo-value approach.
[a] Adjusted for age, gender, cohabitation status, education, and co-morbidity
